# Puerperal Eclampsia

**Published:** 1893-11

**Authors:** Robert Morris

**Affiliations:** Resident Student; Interne


### Clinical Reports.

### CHARITY HOSPITAL CLINICS.

Reported. by Robert Morris, Resident Student.

### PUERPERAL ECLAMPSIA.

It is not proposed to consider the pathology or etiology of puerperal eclampsia, but to simply enumerate the cases that have occurred in 1302 deliveries in the Charity Hospital, and to mention a few special features and the treatment pursued in most cases.

The cases are not sufficiently numerous to be of value as statistics, but they will show that there is a much larger per cent. of puerperal eclampsia in hospital than in private practice. The text-books teach that this condition occurs once in 500 deliveries; in 1302 deliveries in the hospital there were reported 18 cases, making the proportion 1 to 71, instead of 1 to 500. This discrepancy may be due to the fact that the hospital cases, previous to entrance, live under the most unsanitary conditions, such as foul air, poor nourishment, and exposure to the vicissitudes of the weather from lack of clothing, and poor accommodations. Furthermore, no prophylaxis can be instituted, as they have no medical adviser.

These 18 cases consist of 5 post-partum, 7 ante-partum, and 6 ante-post-partum eclampsies. This last division includes those cases in which the convulsions commenced before and continued after delivery. In the ante-partum cases the mortality of the mother was 20%, child 75.8%; in the ante and post-partum, mother 50%, child 80; in post-partum, mother 20, child 65.2%. The average mortality of mother, 30%; child, 65.2%. A great many of the children were non-viable, and this accounts for the large death rate; 86% of the cases occurred in primipara.

The treatment can be more clearly understood by reporting the following case:

M. G., primipara, 21 years of age, was admitted at 9:30 a. m.,

September 18, 1889. She was eight months pregnant. She had had several severe convulsions before admission. At 10:07 a. m. the woman was delivered spontaneously of a still-born child. From 10:40 a. m. to 2:45 p. m., the patient had 29 convulsions, then an interval of two hours, and continued until 11:30 p. m. There were more than thirty-five convulsions. The patient, during this time, was in a deep coma, and remained so until late in the afternoon of the next day, when she regained consciousness, and steadily improved, and was discharged cured a few weeks later.

Urine—September 18th, albumen, 5%; hyaline and granular casts. September 25th, albumen, 10%; no casts. At time of discharge, no albumen, no casts.

Treatment—During convulsions, chloroform was administered. At 11 a. m., September 18th, 20 grains each of chloral and bromide of potassium by rectum, also $\frac{1}{12}$ grain of pilocarpine hydrochlorate by needle; at 11:30 p. m. the third injection of pilocarpine was administered, also 20 grains of bromide and chloral by rectum. The compound jalap powder, in teaspoonful doses, was given on the morning of the 20th, and continued for several mornings. The bromide and chloral was continued three times per day, in 10 grain doses. In no case was bleeding practiced.

In addition to the eclamptic cases, it may be of interest to mention a few cases that presented every evidence of nephritis—œdema, specks before the eyes and albuminous urine with casts, but had no convulsions, or symptoms of impending seizure:

Case 1—A. S. Urine, 35% albumen, granular and hyaline casts. Child, still-born. Mother recovered.

Case 2—M. F., primipara. Urine, 90% of albumen, granular and hyaline casts. Child, still-born. Mother died.

Case 3—R. S., primipara. Urine, 35% of albumen, granular and hyaline casts. Both discharged, well.

Case 4—S. W., primipara. Urine, albumen, 2%, granular and hyaline casts. Child lived. Mother died, seven days after delivery, with suppression of urine.

These cases tend to prove that the relation between puerperal eclampsia and albuminuria is not as intimate as the theory of Lever and Brown would lead you to believe.

### SINGLE KIDNEY.

While holding an autopsy on a man who died from pneumonitis, the abnormality was discovered. The organ occupied the normal position on the left side. It weighed 13 ounces, and was 6 inches in length and 3 inches in width.

Attached to its lower border was a rudimentary kidney, measuring 1¾ inches on convex portion, and 1¼ on concave.

There was one pelvis, and one ureter. The ureter was ¼ inch in width.

